# MedDRA Labeling Groupings to Improve Safety Communication in Product Labels

**DOI:** 10.1007/s43441-022-00393-1

**Published:** 2022-08-08

**Authors:** Ilona Große-Michaelis, Scott Proestel, Radhika M. Rao, Brian S. Dillman, Silvia Bader-Weder, Lynn Macdonald, William Gregory

**Affiliations:** 1 Bayer AG, Research & Development, Pharmaceuticals, Leverkusen, Germany; 2 The U.S. Food and Drug Administration, Center for Drug Evaluation and Research, Silver Spring, USA; 3 AbbVie, Pharmacovigilance and Patient Safety, North Chicago, USA; 4 Eli Lilly and Company, Lilly Research Laboratories, Global Patient Safety – Medical, Indianapolis, USA; 5 F. Hoffmann-La Roche Ltd, Pharma Development, Safety, Basel, Switzerland; 6 Independent, CIOMS MedDRA Labeling Grouping Expert Working Group, Ottawa, Canada; 7 Pfizer, Worldwide Safety and Regulatory, New York, USA

**Keywords:** MedDRA, MedDRA Labeling Groupings (MLG), Product labels, Adverse reactions, ICH

## Abstract

The granularity and structure of the International Council for Harmonisation’s (ICH) Medical Dictionary for Regulatory Activities (MedDRA) are useful for precise coding of adverse events (AEs) for data analysis. In product labeling for healthcare practitioners, however, the granularity of MedDRA Preferred Terms (PTs) can obscure the communication of adverse reactions (ARs). Driven by a focus on patient safety, business needs, and regulatory guidance, many sponsors and regulators have begun to develop institution-specific approaches to clustering similar AR terms in medical product prescribing information on a product-by-product basis. However, there are no agreed upon conventions that describe which AR terms may be appropriate to group together. In order to improve safety communication to patients and healthcare providers, there is an urgent need for a harmonized international approach to the creation and use of groups of MedDRA PTs which we refer to as “MedDRA Labeling Groupings (MLGs)” in medical product prescribing information. Given its long-standing contributions towards the design of Standardised MedDRA Queries (SMQs), the Council for International Organizations of Medical Sciences (CIOMS) convened an Expert Working Group (EWG) with involvement of multiple major stakeholders to produce a consensus document on principles and points to consider in the development of MLGs. The CIOMS MLG EWG identified variations in grouping of MedDRA PTs in product labels, and in the current document, proposes a strategy for improving the communication of drug safety labeling. It is envisaged that the use of these consensus recommendations would be voluntary and applied to product labels in a manner that is consistent with existing regulatory frameworks.

## Background

### The Medical Dictionary for Regulatory Activities (MedDRA) Terminology

The International Council for Harmonisation of Technical Requirements for Pharmaceuticals for Human Use (ICH) is a unique organization that brings together national regulatory authorities and the pharmaceutical industry to discuss scientific and technical aspects of drug registration. As of January 2021, the ICH has united 43 regulatory parties and regional harmonization initiatives, important regional and international industry associations, and other relevant parties [1]. The ICH facilitates and recommends the use of MedDRA through all phases of a medicine’s life cycle, from clinical trials to post-marketing surveillance. The use of MedDRA by all pharmacovigilance stakeholders is required in the European Union (EU) for the “classification, retrieval, presentation in *EU Summary of Product Characteristics (SmPC)*, risk–benefit evaluation and assessment, electronic exchange and communication of pharmacovigilance and medical product information” [2, 3], and is also required by the United States (U.S.) Food and Drug Administration (FDA) for clinical trial safety reporting by all commercial pharmaceutical sponsors [4]. Hence, the Council for International Organizations of Medical Sciences (CIOMS) MedDRA Labeling Groupings (MLGs) Expert Working Group (EWG) proposal for MLG Preferred Term (PT) grouping in medical product information is limited to usage of MedDRA and no other coding terminologies.

The CIOMS MLG EWG envisions that MLGs, which are groupings of near synonymous MedDRA PTs that convey substantially similar clinical concepts, will simplify the product information and enhance safety communication to healthcare providers via the product label. MLGs are intended to communicate an adverse reaction (AR) in a manner that is expected to give the most accurate and understandable description of an adverse reaction in the product label to the healthcare provider.

### Grouping PTs

MedDRA provides several levels of PT groupings, but these can be inadequate to clearly communicate safety information to the healthcare community*.* For labeling purposes, it is generally recommended that ARs be represented as either individual or grouped terms [2, 4, 5].

The MedDRA PTs noted in this manuscript are from MedDRA version 24.0. Due to the high granularity of MedDRA, which has over 24,000 PTs in version 24.0, several PTs may be available within this terminology to represent highly similar clinical concepts. However, groupings within MedDRA, such as High Level Terms (HLTs), can be inadequate to clearly communicate safety information in product labels. For example, the HLT *Gastrointestinal and abdominal pains (excl oral and throat)* includes PTs that might not be appropriate to include in the concept “Abdominal pain,” such as the PTs *Oesophageal pain* and *Visceral pain*. In addition, other parts of the MedDRA hierarchy, such as the HLT *Gastrointestinal signs and symptoms NEC*, contain other PTs that one might want to include with abdominal pain, such as the PT *Abdominal discomfort*. In order to capture relevant PTs from both HLTs, one could consider combining the two HLTs. However, the latter HLT also includes PTs such as *Mastication disorder* and *Hiccups*, which would likely not be considered sufficiently relevant to the concept of abdominal pain.

The need to combine similar PTs was previously discussed by MedDRA’s expert forum called the “Blue Ribbon Panel” in 2005 and subsequently at the ICH MedDRA Management Board. The *ICH MedDRA Data Retrieval and Presentation: Points to Consider* document [6] also discusses the importance of displaying and grouping medically related concepts when presenting estimates of the occurrence of an AR.

The need to group similar ARs is also addressed in regulatory guidance texts [2, 5, 7]. For instance, *U.S. FDA Guidance for Industry* states that “*Events that are reported under different terms in the database, but that represent the same phenomenon (e.g., sedation, somnolence, drowsiness) should ordinarily be grouped together as a single adverse reaction to avoid diluting or obscuring the true effect*” [5]*.* “*Similarly, reactions that represent a syndrome complex should ordinarily be grouped together under an appropriate heading to avoid obscuring the full range of respective symptoms*” [2].

## Building on Past MedDRA Standardization

The principle of grouping PTs that describe similar ARs also applies to searches and presentation of MedDRA-coded data during analysis. The value of Standardised MedDRA Queries (SMQs) in aggregating related PTs into clinically relevant concepts has been shown [8]. Combining the AR terms in medically meaningful ways greatly facilitates the interpretation of data displays in study reports, Integrated Summaries of Safety (ISS), or benefit-risk assessments. These data are subsequently distilled and condensed for communication of important safety concepts to healthcare providers.

A parallel can be drawn between SMQs and the proposed MLGs, as both are intended as tools to promote harmonization and simplification by use of a standardized approach and are developed based on need. The CIOMS MLG EWG’s proposed approach of MedDRA PT grouping of near synonymous terms is anticipated to achieve a clinically meaningful representation of adverse reactions in the product label for healthcare providers. MLGs are not intended to be used for safety signal detection or to alter the approach to safety data analyses.

One SMQ may contain a combination of MedDRA PTs related to a medical concept presenting signs and symptoms, diagnosis, and diagnostic and therapeutic measurements, whereas an MLG is intended to contain MedDRA PTs that are near synonymous to an individual medical concept such as signs or symptoms only or diagnosis only, or diagnostic and therapeutic measurements only. Therefore, an MLG would generally be expected to be contained in an SMQ for the same concept, but not always, due to the requirement that PTs within an MLG are nearly synonymous. However, SMQs would typically be expected to contain many more PTs given its use for safety signal detection and lower threshold for similarity.

However, while SMQs are used for safety signal detection in MedDRA-coded adverse event datasets, MLGs would be used to communicate the ARs in product labeling (Table 1).

**Table 1:** Comparison of SMQ and MLG

Characteristics	SMQ	MLG
Purpose	Safety signal detection	Safety communication in product label
Status	International MedDRA PT grouping that already exists	To be established
Content	Consists of MedDRA PTs that reflect a range or different aspects of a medical concept (Heterogeneous concept)	Consists of MedDRA PTs that are nearly synonymous (Homogeneous concept)
Example	SMQ *Acute renal failure* (narrow scope*) consists of below listed	Proposed MLG *Acute kidney injury* consists of below listed
	19 PTs in MedDRA v24.0:	2 PTs in MedDRA v24.0:
	PT *Acute kidney injury*	PT *Acute kidney injury*
	PT *Acute phosphate nephropathy*	PT *Subacute kidney injury*
	PT *Anuria*	
	PT *Azotaemia*	
	PT *Continuous haemodiafiltration*	
	PT *Dialysis*	
	PT *Foetal renal impairment*	
	PT *Haemodialysis*	
	PT *Haemofiltration*	
	PT *Neonatal anuria*	
	PT *Nephropathy toxic*	
	PT *Oliguria*	
	PT *Peritoneal dialysis*	
	PT *Prerenal failure*	
	PT *Renal failure*	
	PT *Renal failure neonatal*	
	PT *Renal impairment*	
	PT *Renal impairment neonatal*	
	PT *Subacute kidney injury*	

*SMQ *Acute renal failure* broad search consists of 51 PTs (including 19 PTs of narrow scope from above)

## Problem Statement

The CIOMS MLG EWG conducted a brief review using random sampling of several product information (PI) documents from European Public Assessment Reports (EPARs) and the U.S. FDA required labels which demonstrated broad variability in the presentation of MedDRA PT groupings. Although the need for grouping clinically similar ARs in product labels has been recognized, current approaches are not consistent from product to product. Characteristics of grouped PTs vary in the scope of medical concept, severity or etiology. Additionally, for some ARs, similar PTs describing either the clinical diagnosis or the laboratory results (such as “Hyperkalaemia” and “Blood potassium increased”) are variably presented separately or grouped together in product labeling.

Lastly, the MedDRA groupings presented in product labels varied across different regulatory jurisdictions for the same medical product. The CIOMS MLG EWG believes that this finding is due to the absence of conventions or guidelines for grouping PTs, which impairs the accurate representation of safety issues in product labels.

The CIOMS MLG EWG identified a need for globally harmonized principles, for voluntary consideration, to achieve greater consistency and enhanced communication of safety issues in product labels. The CIOMS MLG EWG is also exploring MLG conventions in addition to the harmonized principles listed below to provide more specific guidance for the creation of MLGs.

## Principles of MLGs

The CIOMS MLG EWG recommends the following principles for the development and use of MLGs to improve the clarity of safety information presented in product labels and achieve consistency in presentation of ARs in product labels:

1. MedDRA PTs that convey substantially similar clinical concepts should be combined into MLGs when presented in product labeling.
2. The process of grouping PTs into MLGs should not result in the loss of clinically meaningful safety information.
3. The use of MLGs, while recommended, should be voluntary.
4. The content of MLGs, when used publicly, should be specified in order to ensure transparency.
5. The use of MLGs is intended to foster international harmonization in a manner consistent with existing regulatory frameworks.
6. MLGs should be made easily accessible and widely available to ensure transparency and consistency.

During the creation of these principles, there was extensive discussion regarding the meaning of the following characteristics: (a) “substantially similar clinical concepts”; (b) “transparency”; (c) “easily accessible and widely available”; (d) and minimizing the loss of “clinically meaningful safety information.” These are further discussed below.

The phrase (a) “substantially similar clinical concepts” is referring to the near synonymous nature of the PTs grouped in an MLG. Due to the homogeneity of the near synonymous PTs in an MLG, it is anticipated that one PT will be linked to one MLG only.

The CIOMS MLG EWG was generally in favor of transparency in the application of MLGs to product labeling. However, the concept of (b) “transparency” could be limited to a simple acknowledgment in the product label when MLGs have been used or could refer to a need to specify all of the PTs that have been grouped into an MLG, perhaps as a footnote. Some EWG members favored the latter as it would help readers to have a better understanding of what each MLG represents, while others felt that option might be misleading as it could lead to placing PTs in labels because they exist in an MLG even if they had never occurred in association with the drug. Concern was also raised that listing of the PTs of an MLG in a footnote might unnecessarily complicate the label as the reason the PTs were grouped together in the first place was because of their high degree of similarity. The EWG ultimately decided that this determination would be best made after the creation of MLGs, as the degree of similarity between PTs in the MLGs would be an important factor in the decision.

The concept of (c) “easily accessible and widely available” also received significant attention during the development of the principles. One question raised was whether this concept simply referred to publication of the principles, or whether the expectation would be that whoever ultimately developed the MLGs should ensure their broad dissemination. Concern was also raised as to whether such a principle might be beyond the scope of the EWG and more appropriately left to MLG developer(s). Ultimately, a majority of the EWG believed that as these are intended to be guiding principles, aspirational statements regarding accessibility and availability would be appropriate. It was also noted that having the MLGs “easily accessible and widely available” was consistent with the principle of harmonization, which can only be achieved if the MLGs are readily accessible.

The next concept discussed was the meaning of (d) “clinically meaningful safety information.” The EWG uniformly agreed that MLGs should not result in the loss of clinically meaningful safety information. However, concern was also raised that even the same PT can have a wide range of severities, and the seriousness of PTs is based more on the event outcome than an inherent component of the PT. The EWG decided to keep “clinically meaningful safety information” without further clarification to allow flexibility to the ultimate developers of MLGs. However, in practice, the balance between the benefit of combining similar PTs while also minimizing the loss of clinically meaningful safety information was not always easily achieved when the EWG attempted to create examples of MLGs.

## An MLG Example

“Abdominal pain” is a medical concept commonly reported in product labels. However, multiple other PTs indicate a similar concept, such as the PTs *Abdominal discomfort*, *Epigastric discomfort*, *Gastrointestinal pain*, and *Abdominal tenderness*. Based on the MLG principles, these additional PTs might be grouped into a single MLG called “*Abdominal pain*.” Other PTs, such as *Rebound tenderness* and *Abdominal rigidity*, might not be grouped in such an MLG as they might be considered to represent events with greater severity. Similarly, a PT such as *Oesophageal pain* might be considered more specific than *Abdominal pain* and therefore not included. However, it should also be noted that abdominal pain can be poorly localized by patients, and the location of pain can change over time. Therefore, maintaining the abdominal pain location distinctions made by patients and healthcare providers could potentially be less informative than grouping the PTs together. This is but one of many determinations that will be important for MLG developer(s) to make when they create and maintain MLGs. While creating such standards is not likely to be easy, the CIOMS MLG EWG considers that an open and deliberate process guided by the MLG principles will be a significant advance over the current situation of different institutions taking different approaches when creating their own groupings or not creating groupings at all.

## Challenges and Future Considerations

Once consensus is achieved on the principles, further consistency in product labeling can be achieved if a predefined set of MLGs is created. The EWG acknowledges that this would be a significant undertaking and would require ongoing maintenance of these MLGs with MedDRA versioning. As noted by the principles, such a predefined set of MLGs should be internationally available, easily accessible, and their use transparent to help ensure consistency across regulatory jurisdictions. MLGs are also anticipated to have an impact on other processes that use safety information, e.g., the creation and maintenance of investigator’s brochures, listedness tables in pharmacovigilance, company core data sheets, and safety label information.

## Conclusion

Developing harmonized non-binding principles and guidance for creating MLGs will assist to create more logical and easy to understand safety information for stakeholders. In the longer term, creating a predefined set of non-binding harmonized MLGs could further improve the consistency of communication of ARs in product labels.

Consensus guidelines on MLG principles would not only simplify the approach used across product labels but also contribute to more standardized product-to-product regulatory descriptions, and thus support a meaningful, reproducible, and consistent aggregation of safety information. The groupings could also facilitate considerations in benefit-risk assessments, particularly in instances when modification of product labels is being contemplated.

The CIOMS MLG EWG considers that MLGs created according to the principles that have been proposed in this document will help a wide range of stakeholders. The MLGs will provide a practical and sustainable level of granularity to communicate safety information to the healthcare community globally and consequently contribute to improving the quality of medical treatment that patients receive.
